# Should We be Concerned with Nicotine in Sport? Analysis from 60,802 Doping Control Tests in Italy

**DOI:** 10.1007/s40279-023-01819-y

**Published:** 2023-02-24

**Authors:** Thomas Zandonai, Francesco Botrè, Maria Gabriella Abate, Ana María Peiró, Toby Mündel

**Affiliations:** 1 Department of Pharmacology, Paediatrics and Organic Chemistry, Miguel Hernández University of Elche, Crta. Nacional, N-332. S/N, Sant Joan, 03550 Alicante, Spain; 2 Neuropharmacology on Pain and Functional Diversity (NED), Institute of Health and Biomedical Research of Alicante (ISABIAL Foundation), Alicante, Spain; 3 Department of Social and Developmental Psychology, “Sapienza” University of Rome, Rome, Italy; 4 Laboratorio Antidoping, Federazione Medico Sportiva Italiana, Rome, Italy; 5 REDs, Research and Expertise on Anti-Doping Sciences, ISSUL Institute des Sciences du Sport, University of Lausanne, Lausanne, Switzerland; 6 Pain Unit, Department of Health of Alicante-General Hospital, Alicante, Spain; 7 Clinical Pharmacology Unit, Department of Health of Alicante, General Hospital, Alicante, Spain; 8 School of Sport, Exercise and Nutrition, Massey University, Palmerston North, New Zealand; 9 Department of Kinesiology, Brock University, St. Catharines, Canada

## Abstract

**Background:**

Nicotine is a psychostimulant drug with purported use in sports environments, though the use of nicotine among athletes has not been studied extensively.

**Objective:**

The aim of this study was to assess the nicotine positivity rate in 60,802 anti-doping urine samples from 2012 to 2020.

**Methods:**

Urine samples obtained in-competition at different national and international sports events held in Italy during the period 2012–2020 were analysed. All samples were from anonymous athletes that were collected and analysed at the WADA-accredited antidoping laboratory in Rome, Italy. Samples were analysed by gas chromatography coupled with mass spectrometry, with a cut-off concentration for nicotine of > 50 ng/mL. Results were stratified by year, sport and sex.

**Results:**

An overall mean of 22.7% of the samples (*n* = 13,804; males: *n* = 11,099; females: *n* = 2705) showed nicotine intake, with male samples also displaying higher positivity rates than female (24.1% vs 18.5%). Sample positivity was higher during 2012–2014 (25–33%) than 2015–2020 (15–20%). Samples from team sports displayed a higher positivity rate than those from individual sports (31.4 vs 14.1%).

**Conclusions:**

The current data demonstrates that one in five samples from a range of 90 sports test positive for nicotine in-competition. There is a lower positivity rate in endurance versus power/strength athletes and higher positivity rate in team versus individual sports, probably accounted for by differences in physiological and psychological demands and the desire for socialisation. WADA, international and national sports federations should consider these findings with concern, proactively investigate this phenomenon and act in order to protect the health and welfare of its athletes.

**Supplementary Information:**

The online version contains supplementary material available at 10.1007/s40279-023-01819-y.

## Key Points

**Table:** 

WADA currently has nicotine on its Monitoring Program due to high use in winter sports, although current research evidence does not support a clear ergogenic effect.
This study highlights that one in five samples from a range of 90 sports test positive for nicotine in-competition, with use amongst aerobic/endurance athletes greatest, followed by team sports.
International and national sports federations should proactively investigate this phenomenon and act in order to protect the health and welfare of its athletes.
WADA should continue to monitor nicotine use and support research that further determines user trends to understand why and how athletes use nicotine.

## Introduction

After caffeine, nicotine is the most widely consumed psychoactive substance in the world [1], with a prevalence of consumption in excess of 20% for the global population [2] in the form of smoked and smokeless tobacco, and a range of products marketed as nicotine replacement therapies. In the sporting domain, whilst nicotine consumption through the use of chewing tobacco became synonymous with professional baseball [3], it was the discovery of its high use amongst winter sports [4, 5] that led to the World Anti-Doping Agency (WADA) placing nicotine on its Monitoring Program in 2012 [6]. This signalled that WADA wanted to detect patterns of (mis)use to determine whether nicotine could enhance sport performance or increase the health risk to an athlete that would see it upgraded to the List of Prohibited Substances.

Athletes believe the consumption of nicotine (or related substances) to be beneficial by preventing a dry mouth through increased saliva secretion, stimulating satiety to control body weight, improving concentration and reaction time, and helping relaxation to achieve a desirable arousal-attention (for review, see [3]). Despite exerting physiological and psychological effects that should be ergogenic and nootropic, the current evidence base does not support this [3]. However, long-term exposure to nicotine (i.e. chronic users) could lead to addiction that develops tolerance, withdrawal and dependence [7], whereby nicotine use in this cohort has been shown to improve exercise performance when nicotine-abstinent compared with -satiated [8].

Given the health risks to athletes posed by long-term use of nicotine directly, and indirectly through tobacco [9], and that research on this topic has not been forthcoming in recent years, the purpose of this paper was to describe the positivity rate of nicotine use in athletes and determine any temporal and user trends from a sample of 60,802 doping control tests from 2012 to 2020.

## Materials and Methods

### Sample Collection

All urine samples were collected from national and international in-competition (IC) doping control tests that took place in Italy between 2012 and 2020. Note, sample collection (and analyses) for 2020 were adversely affected by the emergence of COVID-19. Samples were anonymously analysed as part of the WADA Monitoring Program at the WADA-accredited antidoping laboratory in Rome, Italy. The sample was composed of national and international athletes that were tested during sports competitions held in Italy. According to the WADA Code, we analysed urine samples collected from participants that were regularly enrolled in the national and international sports federations of each discipline (elite and amateur levels and paralympic sports).

### Sample Analysis

Concerning the techniques employed in the analysis of the samples, we used gas chromatography (GC) coupled with mass spectrometry (GS/MS) with electronic ionisation and acquisition in selected ion monitoring. The GC/MS system was a GC 7890 from Agilent Technologies interfaced with an Agilent 5975 MS, with an HP5 column (17 m × 0.2 mm × 0.33 μm). The parameters for GC were the following: oven programme – 85 °C (1 min), 15 °C/min to 270 °C, 50 °C/min to 310 °C (3.5 min); injection – 1 µL, pulsed splitless; injector temperature – 270 °C. We followed the protocol that is normally used for narcotics and stimulants [10]. The urine samples were alkalinised with NaOH and NaCl was added for a salting out effect. The samples were extracted with *tert*-butyl methyl ether. The organic extracts were then taken to dryness under a reduced nitrogen flow at room temperature and reconstituted with an extraction solvent before GC/MS analysis. Diphenylamine was added as an internal standard (ISTD).

The estimated nicotine concentration was > 50 ng/mL according to the WADA monitoring program, which is based on allowing for the lower limit of quantification and a conservative concentration limit for active consumption [4]. The advantage of this conservative limit is that active consumption is clearly distinct from passive exposure (e.g. tobacco smoke) and trace amounts through diet (e.g. potatoes, cauliflower) in ‘tobacco-free’ individuals, as well as abstinent nicotine users [3, 4]. However, a disadvantage is a potential under-estimation of true positivity.

### Data Analysis and Statistics

Data were presented as number of urine samples, number of nicotine (positive) samples and stratified by biological sex using positivity rate (percentage). We further stratified positivity (percentage) by year and for team and individual sports. All data management and analyses were performed using GraphPad Prism (GraphPad Software, Inc., CA, USA).

## Results

**Fig. 1:**
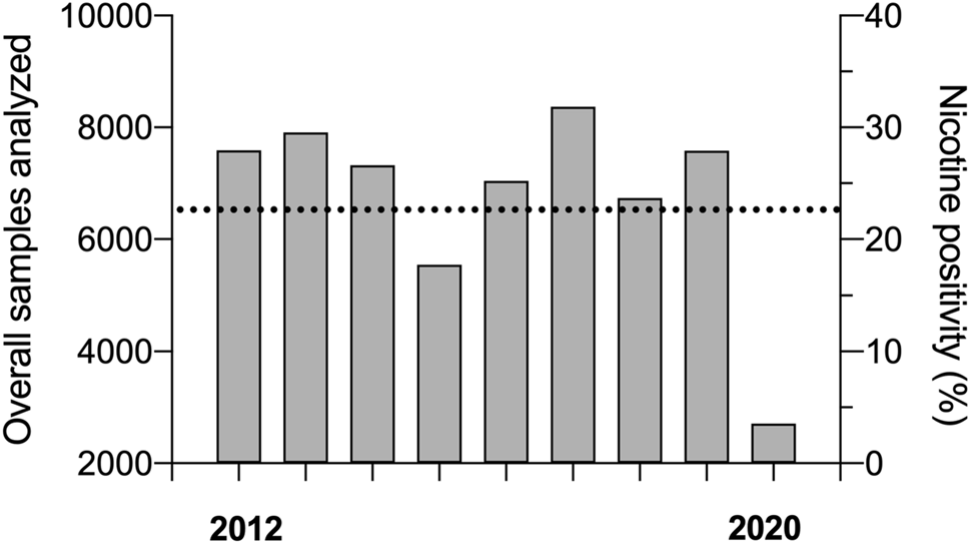
Total samples in competition by year with detected nicotine positivity (%). Bars represent total sample for each year; horizontal black dashed line represents the overall mean for nicotine positivity (22.7%)

**Fig. 2:**
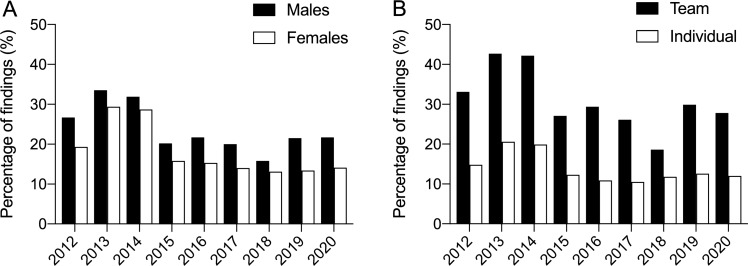
Positivity rate (%) of nicotine for males and females (**A**) and team and individual sports (**B**)

Figure 1 displays that 13,804 samples showed nicotine intake (males = 11,099 [overall mean 24.1%]; females = 2705 [overall mean 18.5%]), equating to an overall mean positivity of 22.7%. As can be seen in Fig. 1 and Table 1, there was a decline in positivity rate in 2015 that was maintained until 2020. Of 90 sports, 83 (92.2%) showed at least one sample that contained nicotine (Supplementary Table 1, see electronic supplementary material [ESM]). The most represented sport was association football (soccer) with 20,454 tests, with overall nicotine positivity at 29% (range from 18 to 40% depending on year). Figure 2A displays trends for positivity in male and female samples, while Fig. 2B shows team (overall mean 31.4%) and individual (overall mean 14.1%) sports. Table 2 further details sports above the sample median per year, and shows that 90.1% (73/81) of team sports compared with 39.9% of individual sports (69/173) exhibited nicotine use greater than the 20% global consumption in the general population. Table 2 also shows that athletes in endurance events/sports displayed a lower rate of positivity than those engaged in power and strength sports/disciplines. This is highlighted particularly well by the results from athletics (Supplementary Table 2, see ESM), the third most represented sport.

**Table 1:** Nicotine positivity rate in all samples analysed

Year	Urine samples			Nicotine positive samples			Nicotine positivity %
	Total (*N*)	Males (*N*)	Females (*N*)	Total (*N*)	Males (*N*)	Females (*N*)	
2012	7591	5898	1693	1901	1575	326	25.0
2013	7909	6034	1875	2574	2022	552	32.5
2014	7321	5555	1766	2277	1771	506	31.1
2015	5540	4346	1194	1069	880	189	19.3
2016	7042	5357	1685	1421	1164	257	20.2
2017	8368	6330	2038	1550	1265	285	18.5
2018	6737	5180	1557	1023	819	204	15.2
2019	7582	5376	2206	1450	1154	296	19.1
2020	2712	2073	639	539	449	90	19.9

**Table 2:** Nicotine positivity collected in-competition from 2012 to 2020 by sport

	2012		2013		2014		2015		2016		2017		2018		2019		2020	
	%	*n*	%	*n*	%	*n*	%	*n*	%	*n*	%	*n*	%	*n*	%	*n*	%	*n*
Team sports																		
American football	41.7	36	41.7	36											41.8	55		
Baseball	74.7	75	65.0	60	85.5	55	47.8	46	47.1	51	55.8	43			66.7	72		
Basketball	37.2	309	40.4	230	47.0	304	32.1	190	27.2	393	22.5	396	16.6	308	24.6	289	17.6	85
Handball	47.5	80	61.3	142	71.1	54	46.3	54	38.9	131	29.7	155	18.9	74	29.5	61	23.3	30
Hockey (field)	37.7	61	51.9	27	45.5	33			28.7	101			10.0	40				
Hockey (ice)	46.3	82	61.3	88					53.8	80	34.3	70	18.1	72	39.3	56	44.4	36
Hockey (indoor)	30.0	40	35.2	37	40.0	30			48.5	132	33.7	95						
Rugby	38.0	184	46.8	109	45.5	99	17.7	175	32.1	187	30.5	174	17.4	201	34.8	282	25.0	60
Soccer	31.3	3003	40.4	3078	39.0	2551	26.0	1943	26.2	1926	25.1	2879	18.4	2325	27.3	1623	26.1	1126
Volleyball	32.1	249	53.6	248	46.4	265	31.7	145	33.9	274	27.0	237	23.8	261	37.4	222	39.5	124
Water polo	21.5	191	38.5	252	40.2	209	23.6	140	21.5	261	25.9	239	21.5	135	21.4	187	34.6	26
Individual sports																		
Archery			30.6	49	68.1	47	27.9	37			27.6	86	8.7	46	29.0	62	52.6	19
Athletics	9.9	476	15.8	704	12.4	676	5.7	488	7.2	636	6.1	865	12.6	540	6.8	851	3.7	216
Biathlon													8.3	48				
Body building											20.8	72						
Boxing	15.0	160	32.3	124	36.3	135	13.5	133	6.7	90	10.8	93	9.0	111	18.6	156	14.5	62
Canoe	4.5	44	8.3	48	11.4	79	6.3	63					4.9	82	11.8	85		
Climbing					13.5	37	7.4	27							12.7	72		
Cycling	5.6	948	8.2	1067	8.5	1135	4.9	896	4.0	1048	5.2	1052	9.3	783	4.6	862	2.3	260
Dance sports					57.1	42											25.0	16
Diving	34.4	32			43.1	51			43.6	39	34.5	58	25.5	55	26.2	65		
Equestrian							56.7	30	23.6	55							47.1	17
Fencing	42.1	159	47.1	121	43.3	120	27.3	55	44.9	49	21.6	116	15.7	108	26.2	122		
Golf	34.8	46			50.0	36												
Gymnastics	22.4	58	20.4	54	25.8	62	26.0	50	20.9	43	4.9	81	22.8	57	26.0	131	28.1	32
Judo	7.7	65	18.4	38			18.4	38	11.9	59	15.7	51			17.9	140		
Karate																	31.3	16
Kickboxing													10.8	51				
Lifesaving											8.2	61	6.7	45				
Motorcycling racing																	15.8	19
Powerlifting																	25.0	24
Roller sports									8.9	45	15.2	66					6.3	16
Rowing			9.0	67	8.3	96	3.2	63	8.0	75	7.3	110	3.6	56	6.3	143		
Sailing					17.2	29												
Shooting	34.3	108	42.9	28	33.3	69	40.0	55	26.0	96	28.6	42			28.2	163	38.9	18
Skating (artistic)			33.3	36														
Skating (speed)			27.3	161	3.6	28	14.3	35			10.6	104			9.6	157		
Skiing (alpine)	5.4	93	58.3	65	57.8	64			25.4	106	27.8	54	16.6	73	16.5	97	12.5	24
Skiing (cross c.)	15.3	72	23.3	90	33.9	56					13.3	60	10.2	49	14.0	86		
Skiing (mountaineer)			19.6	46	25.0	32												
Swimming	10.1	149	10.8	158	16.7	239	14.2	219	6.4	362	2.5	322	9.1	350	5.7	420	1.9	106
Taekwondo			33.3	27											17.7	62		
Tennis	29.2	106	32.8	64	18.2	77	23.4	64	14.3	91	13.3	75			15.4	65		
Tennis (table)	14.0	43	40.0	45														
Triathlon	6.3	79	3.3	60	3.6	83			3.6	84	5.7	88	7.8	115	1.7	181		
Underwater sports	5.3	38	22.2	27	22.6	53			6.8	103			13.6	66	10.3	58		
Weightlifting	47.4	38	60.0	50			56.7	30	40.3	67	38.6	83	19.8	121	39.4	66		
Wrestling			31.0	29			45.2	31					10.8	83	15.5	84	12.5	72

Data are presented as positivity rate of nicotine (> 50 ng/ml) present in urine samples in percentage values (%) and number of samples analysed (*n*) in competitions stratified by team sports and individual sports. The sample cut-off is the median for each year (2012: 36; 2013: 27; 2014: 27; 2015: 27; 2016: 39; 2017: 40; 2018: 40; 2019: 55; 2020: 16)

## Discussion

The novel and important results of this investigation are that (i) one in five samples from a range of 90 sports contained nicotine in-competition, (ii) there is a trend of declining consumption across the nine years of anti-doping urinalysis, (iii) samples from team sports displayed approximately twice the positivity than those from individual sports, and (iv) one in four male and one in five female samples showed nicotine positivity. The current data supports previous doping control reports [4, 5] in demonstrating a high positivity rate of active nicotine use by athletes in-competition that is sustained temporally and across many sports.

### Strong User Trends are Demonstrated for Nicotine Use

We observed a clear difference in nicotine use between different sports. Table 2 highlights that in general, athletes requiring high aerobic capacity (endurance, e.g. cycling, athletics, triathlon, rowing, swimming) displayed much lower nicotine use than those more reliant on strength and power (e.g. American football, baseball, weightlifting) and skill and tactics (e.g. golf, fencing, shooting). This observation is similar to previous reports from athletes [11, 12] and is best explained by the fact that tobacco use could reduce aerobic and muscular performance, which would lead to lower consumption [3], and/or that endurance athletes have greater health awareness of the long-term use of nicotine/tobacco [9]. Future research could include more detailed analysis around this matter by using, for example, a standardised framework that provides quantitative assessment of the demands across different sports that are comparable in their athletic variables (i.e. bioenergetic, neuromuscular, psychomotor and biomechanical factors) to group sports as *strength and power, muscular endurance, aerobic endurance, movement agility, reaction time, psychomotor skill and accuracy* [13]*.*

It was surprising to observe a high positivity in sports with high overall metabolic demand such as basketball, handball, hockey, association (soccer) and rugby football, and volleyball (Table 2). As suggested by McDuff et al. [14], such use is more likely a result of the social environment and/or reflects patterns of use that are explained not by a drive for performance-enhancement but by the need for relaxation and/or recovery. For example, an investigation into snus use by footballers in the UK revealed an alarming and increasing trend [15]. One high-profile player, Jamie Vardy, admitted snus “…*helped me chill out…*” [15], something that is corroborated by practitioners who claim widespread practice, with players using and sharing products openly [16]. This type of use may be explained by the fact that at high doses nicotine exerts a depressant or relaxant effect through the activation of endogenous opioid pathways [17], whilst nicotine could increase the pain threshold by exerting antinociceptive effects [18, 19]. Data from winter sports indicate that in addition to high use [4, 5], regular smokeless tobacco use induces greater satisfaction and psychological reward, similar to the effects of smoking tobacco [20]. A recent study showed that athletes who practiced high-intensity sports were less likely to smoke cigarettes compared with low-intensity sport athletes. Moreover, those practicing an individual sport are less likely to use smokeless tobacco and more likely to vape compared with those practicing team sports [21]. Team sports were also associated with increased use of smokeless and smoked tobacco compared with no participation in team sports [22], confirming, as in our sample, that socialisation could be fundamental in this type of behaviour. Notably, our data supports an increased use in team compared with individual sports (Fig. 2B). However, this is likely secondary to aerobic demand, as discussed above, as some individual sports (e.g., archery, diving, fencing, shooting, weightlifting) consistently displayed high use (Table 2).

### Research and Policy Implications

Let us use association football (soccer) as an example, as this represented the largest cohort within our sample (Table 2; *n* = 20,454 or 34%). Overall positivity of nicotine amongst this cohort was 29% (yearly range 18–40%) with the high-profile investigation noted above uncovering the extent of this problem. Professional football players display elevated levels of supplement use—some without seeking advice—but low levels of knowledge about doping, with a majority not expecting to be tested for drugs [23, 24]. This type of behaviour makes it imperative that national and international governing bodies proactively investigate this concerning issue and act to protect the health and welfare of its athletes, for example with anti-doping prevention and education programmes [24]. Additionally, results from the current study and previous doping control reports [4, 5] should convince WADA to maintain nicotine’s inclusion in its monitoring programme to further determine patterns of use amongst athletes that would afford a better understanding of why and how athletes use nicotine; this could inform policy or interventions to reduce consumption and minimise harm.

Nicotine is highly addictive and poses health risks to athletes, especially through long-term use, whether caused by nicotine directly or indirectly through tobacco [9]. The current data indicate that since 2012, nicotine use amongst athletes has declined, although Fig. 1 shows that since 2015 positivity has been sustained. This mirrors declining global trends in tobacco use, for both males and females, because of tobacco control measures [25]. Nevertheless, there is still cause for concern given that for young adults and youths, > 70% of nicotine consumption is attributable to vaping [26]. There is a distinction between risk reduction from tobacco cessation and potential harm from nicotine dependence and the gateway effect, whereby nicotine replacement therapies occupy a lower place on the harm continuum than tobacco; they are not harmless or risk-free [26–28]!

### Limitations

The current data set is taken from one country only, and therefore may not be representative of other countries or regions. However, as an example, trends for tobacco use in Italy mirror those globally, both in terms of prevalence and declining rate [25, 29]. Further, the current larger data set is not dissimilar to that reported from the Swiss Laboratory for Doping Analyses 2010–11 [5]. Adopting a conservative concentration limit for the presence of urinary nicotine may underestimate true positivity but allows determination of active consumption (cf. passive smoking). However, anti-doping analyses such as this do not allow the route of nicotine consumption to be identified (i.e. smoked vs smokeless tobacco vs nicotine replacement products [electronic cigarettes]). Such information is necessary in order to understand why and how athletes use nicotine, in an attempt to reduce consumption and minimise harm. Finally, it was not possible to calculate (period) prevalence with the current data set due to the following: no baseline data, knowledge of true random testing, unique versus duplicate athlete sampling, the wider population (i.e. Italian vs international athletes). However, the fact that our positivity rates are in line with previous research [4, 5, 11, 12, 21, 22] provides confidence.

## Conclusion

The finding of 22.7% nicotine positivity among samples is alarming and should not be underestimated. Our findings should be considered with concern by stakeholders in the sports environment. They should further investigate this phenomenon and act in order to protect the health and welfare of their athletes.

## Supplementary Information

Below is the link to the electronic supplementary material.

Supplementary file1 (PDF 442 KB)

Supplementary file2 (PDF 50 KB)

## Declarations

### Availability of data and material

All data that support the findings of this study are shown in the Supplementary Material Table 1 (see electronic supplementary material).
