# Supplementary material for: Should We be Concerned with Nicotine in Sport? Analysis from 60,802 Doping Control Tests in Italy
Source: Sports Med. 2023 Feb 24;53(6):1273–9. doi: 10.1007/s40279-023-01819-y (PMC9951140; doi:10.1007/s40279-023-01819-y)
Supplement: Supplementary file 1 — Supplementary file1 (PDF 442 KB) [file 40279_2023_1819_MOESM1_ESM.pdf]

Should we be concerned with nicotine in sport?

Analysis from 60,802 doping control tests in Italy

Thomas Zandonai*[1,2,3], Francesco Botrè[4,5], Maria Gabriella Abate[4], Ana María Peiró[2,6,7], Toby Mündel[8,9]

[1] Department of Pharmacology, Paediatrics and Organic Chemistry Miguel Hernández University of Elche, Alicante, Spain

[2] Neuropharmacology on Pain and Functional Diversity (NED), Institute of Health and Biomedical Research of Alicante (ISABIAL Foundation), Alicante, Spain

[3] Department of Social and Developmental Psychology "Sapienza" University of Rome, Rome, Italy

[4] Laboratorio Antidoping, Federazione Medico Sportiva Italiana, Rome, Italy

[5] REDs - Research and Expertise on anti-Doping Sciences, ISSUL Institute des Sciences du Sport, University of Lausanne, Lausanne, Switzerland

[6] Pain Unit, Department of Health of Alicante-General Hospital, Alicante, Spain

[7] Clinical Pharmacology Unit, Department of Health of Alicante - General Hospital, Alicante, Spain

[8] School of Sport, Exercise and Nutrition, Massey University, Palmerston North, New Zealand

[9] Department of Kinesiology, Brock University, St. Catharines, Canada

Thomas Zandonai: ORCID https://orcid.org/0000-0002-7606-9675

Twitter: @thomaszando

Francesco Botré: ORCID https://orcid.org/0000-0001-5296-8126

Twitter: @Botrek

Ana María Peiró: ORCID https://orcid.org/0000-0002-2385-3749

Twitter: @ampeiro

Toby Mündel: ORCID https://orcid.org/0000-0002-4214-8543

Twitter: @kiwiheatlab

Corresponding author:

Thomas Zandonai, PhD

Department of Pharmacology, Pediatrics and Organic Chemistry, Miguel Hernández University of Elche

Alicante, Spain

Address: Crta. Nacional, N-332. s/n, 03550 Sant Joan, Alicante, Spain

E-mail address: tzandonai@umh.es

Supplementary Material Table 1.

Nicotine positivity collected In Competition from 2012 to 2020 in all sports.

Year	Total Test	Nicotine cases	Nicotine %
2012	7591	1901	25,0

SPORTS	TEST	Males Test	Nicotine Males	Females Test	Nicotine Females	Total Nicotine	Nicotine %
Soccer	3003	2809	875	194	65	940	31,3
Cycling	948	783	47	165	6	53	5,6
Athletics	476	277	26	199	21	47	9,9
Basketball	309	216	82	93	33	115	37,2
Volleyball	249	111	38	138	42	80	32,1
Water Polo	191	92	23	99	18	41	21,5
Rugby	184	184	70	0	0	70	38,0
Boxing	160	148	17	12	7	24	15,0
Fencing	159	82	39	77	28	67	42,1
Swimming	149	77	10	84	6	16	10,7
Skating Speed	117	63	5	54	2	7	6,0
Shooting	108	67	29	41	8	37	34,3
Tennis	106	68	24	38	7	31	29,2
Skiing Alpine	93	51	4	42	1	5	5,4
Hockey Ice	82	80	38	2	0	38	46,3
Handball	80	40	23	40	15	38	47,5
Triathlon	79	49	4	30	1	5	6,3
Baseball	75	75	56	0	0	56	74,7
Skiing Cross Country	72	35	11	37	0	11	15,3
Judo	65	33	2	32	3	5	7,7
Hockey Field	61	48	17	13	6	23	37,7
Gymnastics	58	18	8	40	5	13	22,4
Golf	46	22	9	24	7	16	34,8
Canoe/Cayak	44	24	0	20	2	2	4,5
Tennis Table	43	33	6	10	0	6	14,0
Hockey Indoor	40	40	12	0	0	12	30,0
Underwater Sports	38	27	1	11	1	2	5,3
Weightlifting	38	22	8	16	10	18	47,4
American Football	36	36	15	0	0	15	41,7
Archery	32	20	4	12	4	8	25,0

Diving	32	15	7	17	4	11	34,4
Karate	31	13	4	18	4	8	25,8
Life Saving	27	20	3	7	0	3	11,1
**Climbing**	26	21	11	5	1	12	46,2
Wrestling	26	18	3	8	3	6	23,1
Taekwondo	24	12	3	12	3	6	25,0
Softball	22	0	0	22	5	5	22,7
Modern Pentathlon	17	7	1	10	0	1	5,9
Equestrian	16	10	7	6	3	10	62,5
Powerboating	14	14	5	0	0	5	35,7
Kickboxing	13	8	4	5	2	6	46,2
Sailing	13	10	3	3	1	4	30,8
Motorcycle Racing	12	12	5	0	0	5	41,7
Automobile Sports	11	11	1	0	0	1	9,1
Bodybuilding	10	5	1	5	1	2	20,0
Water Skiing	10	7	2	3	0	2	20,0
DanceSport	9	5	2	4	0	2	22,2
Kendo	6	4	2	2	0	2	33,3
Bowling	5	5	1	0	0	1	20,0
Dragon Boat	5	4	0	1	1	1	20,0
Squash	5	3	1	2	0	1	20,0
Casting	4	4	1	0	0	1	25,0
Full contact	4	4	1	0	0	1	25,0
Parachuting	4	4	3	0	0	3	75,0
Balle au Tambourin	2	2	1	0	0	1	50,0
**TOTAL**	**7489**	**5848**	**1575**	**1653**	**326**	**1901**	
**Median**	**36**						

**NO Nicotine**	**TEST**	Males	Females
Badminton	4	2	2
Basque Pelota	2	2	0
Biathlon	20	10	10
Bridge	2	2	0
Chess	4	4	0

Cricket	2	2	0
Draughts	4	0	4
Hunting sports	2	2	0
Roller Sports	4	2	2
Rowing	40	22	18
Skating Artistic	8	4	4
**TOTAL**	**92**	**52**	**40**

Year	TEST Sports	Nicotine	%
2013	7909	2574	32,5

SPORTS	TEST	Males Test	Nicotine Males	Females Test	Nicotine Females	Total Nicotine	Nicotine %
Soccer	3078	2858	1125	220	120	1245	40,4
Cycling	1067	866	78	201	10	88	8,2
Athletics	704	384	57	320	54	111	15,8
Water Polo	252	116	48	136	49	97	38,5
Volleyball	248	128	73	120	60	133	53,6
Basketball	230	200	81	30	12	93	40,4
Skating Speed	169	85	15	84	29	44	26,0
Swimming	158	76	6	82	11	17	10,8
Handball	142	84	50	58	37	87	61,3
Boxing	124	114	40	10	0	40	32,3
Fencing	121	62	29	59	28	57	47,1
Rugby	109	109	51	0	0	51	46,8
Skiing Cross-Country	90	48	18	42	3	21	23,3
Hockey Ice	88	80	50	8	2	52	59,1
Rowing	67	47	5	20	1	6	9,0
Skiing Alpine	65	36	22	29	13	35	53,8
Tennis	64	41	14	23	7	21	32,8
Baseball	60	60	39	0	0	39	65,0
Triathlon	60	35	1	25	1	2	3,3
Gymnastics	54	13	6	41	5	11	20,4
Weightlifting	50	30	14	20	16	30	60,0
Archery	49	29	9	20	6	15	30,6
Canoe/Kayak	48	32	2	16	2	4	8,3
Skiing Mountaineering	46	26	6	20	3	9	19,6
Tennis Table	45	28	13	17	5	18	40,0
Judo	38	11	3	27	4	7	18,4
Hockey Indoor	37	27	9	10	4	13	35,1
American Football	36	36	15	0	0	15	41,7
Skating Artistic	36	17	10	19	2	12	33,3
Wrestling	29	20	8	9	1	9	31,0

Shooting	28	16	8	12	4	12	42,9
Hockey Field	27	17	6	10	8	14	51,9
Taekwondo	27	14	4	13	5	9	33,3
Underwater Sports	27	21	4	6	2	6	22,2
Lifesaving	25	12	4	13	3	7	28,0
Skiing Freestyle	25	13	6	12	7	13	52,0
Dance Sport	24	12	5	12	5	10	41,7
Karate	23	13	5	10	1	6	26,1
Softball	23	4	2	19	7	9	39,1
Biathlon	22	11	0	11	1	1	4,5
Equestrian	22	14	7	8	4	11	50,0
Golf	22	13	7	9	1	8	36,4
Diving	21	10	4	11	4	8	38,1
Sailing	21	10	3	11	2	5	23,8
Squash	18	12	5	6	6	11	61,1
Climbing	17	9	2	8	0	2	11,8
Kickboxing	16	15	9	1	1	10	62,5
Snowboard	16	8	2	8	1	3	18,8
Waterskiing	12	8	4	4	2	6	50,0
Motorcycle Racing	11	11	4	0	0	4	36,4
Muaythai	11	8	3	3	1	4	36,4
Orienteering	11	8	6	3	0	6	54,5
Automobile Sports	8	7	2	1	0	2	25,0
Casting	8	8	6	0	0	6	75,0
Badminton	6	4	1	2	1	2	33,3
Bodybuilding	6	4	2	2	0	2	33,3
Bowling	6	6	6	0	0	6	100,0
Powerboating	6	6	2	0	0	2	33,3
Air Sports	3	3	1	0	0	1	33,3
Chess	3	3	2	0	0	2	66,7
Balle au Tambourin	2	0	0	2	1	1	50,0
Bridge	2	2	1	0	0	1	50,0
Cricket	2	2	1	0	0	1	50,0
Hunting sports	2	2	1	0	0	1	50,0

**TOTAL**	**7867**	**6004**	**2022**	**1863**	**552**	**2574**
**Median**	**27**					

**NO Nicotine**	**TEST**	Males	Females
Diving	1	1	0
Basque Pelota	2	2	0
Boules Sports	3	3	0
Curling	4	2	2
Draughts	3	3	0
Modern Pentathlon	9	4	5
Rafting	2	2	0
Skiing Nordic Combined	16	13	0
Twirling	2	0	2
**TOTAL**	**42**	**30**	**9**

Year	TEST Sports	Nicotine	%
2014	7321	2277	31,1

SPORTS	TEST	Males Test	Nicotine Males	Females Test	Nicotine Females	Total Nicotine	Nicotine %
Soccer	2551	2421	928	130	67	995	39,0
Cycling	1135	972	83	163	13	96	8,5
Athletics	676	404	52	272	32	84	12,4
Basketball	304	231	101	73	42	143	47,0
Volleyball	265	99	58	166	65	123	46,4
Swimming	239	129	21	110	19	40	16,7
Water Polo	209	93	46	116	38	84	40,2
Boxing	135	93	31	42	18	49	36,3
Fencing	120	58	27	62	25	52	43,3
Rugby	99	99	45	0	0	45	45,5
Rowing	96	54	5	42	3	8	8,3
Handball	83	45	33	38	26	59	71,1
Triathlon	83	57	1	26	2	3	3,6
Canoe/Kayak	79	42	8	37	1	9	11,4
Tennis	77	64	9	13	5	14	18,2
Shooting	69	45	15	24	8	23	33,3
Skiing Alpine	64	41	25	23	12	37	57,8
Gymnastics	62	20	10	42	6	16	25,8
Skiing Cross-Country	56	28	15	28	4	19	33,9
Baseball	55	55	47	0	0	47	85,5
Underwater Sports	53	33	6	20	6	12	22,6
Diving	51	24	11	27	11	22	43,1
Archery	47	33	23	14	9	32	68,1
Dance Sport	42	20	9	22	15	24	57,1
Climbing	37	23	4	13	1	5	13,5
Golf	36	22	10	14	8	18	50,0
Hockey Field	33	16	6	17	9	15	45,5
Skiing Mountaineering	32	16	5	16	3	8	25,0
Hockey Indoor	30	30	12	0	0	12	40,0
Sailing	29	20	5	9	0	5	17,2

Skating Speed	28	14	1	14	0	1	3,6
Hockey Ice	26	22	16	4	1	17	65,4
Skating Aristic	26	7	3	19	4	7	26,9
Judo	25	7	0	18	3	3	12,0
Weightlifting	25	12	5	13	9	14	56,0
Wrestling	25	16	8	9	1	9	36,0
Softball	24	0	0	24	10	10	41,7
Karate	23	11	1	12	6	7	30,4
Kickboxing	22	13	9	9	3	12	54,5
Tennis Table	21	10	5	11	2	7	33,3
Waterskiing	20	13	10	7	0	10	50,0
Lifesaving	18	6	3	12	3	6	33,3
American Football	16	16	11	0	0	11	68,8
Automobile Sports	15	15	4	0	0	4	26,7
Squash	15	10	7	5	0	7	46,7
Taekwondo	15	9	4	6	5	9	60,0
Equestrian	12	6	5	6	3	8	66,7
Roller Sports	12	4	3	8	0	3	25,0
Modern Pentathlon	10	5	0	5	1	1	10,0
Powerboating	8	8	5	0	0	5	62,5
Bodybuilding	7	4	2	3	2	4	57,1
Motorcycle Racing	7	7	1	0	0	1	14,3
Biathlon	6	3	1	3	0	1	16,7
Dragon Boat	6	3	1	3	1	2	33,3
Skiing Freestyle	6	3	3	3	1	4	66,7
Snowboard	6	3	1	4	2	3	50,0
Balle au Tambourin	4	2	1	2	1	2	50,0
Bridge	4	4	2	0	0	2	50,0
Full contact	4	4	2	0	0	2	50,0
Rafting	4	4	2	0	0	2	50,0
Tug of War	4	4	2	0	0	2	50,0
Bowling	3	3	2	0	0	2	66,7
**TOTAL**	**7294**	**5535**	**1771**	**1759**	**506**	**2277**	
**Median**	**27**						

**NO Nicotine**	**TEST**	Males	Females
Casting	10	10	0
Duathlon	3	3	0
Orienteering	14	7	7
**TOTAL**	**27**	**20**	**7**

Year	TEST Sports	Nicotine	%
2015	5540	1069	19,3

SPORTS	TEST	Males Test	Nicotine Males	Females Test	Nicotine Females	Total Nicotine	Nicotine %
Soccer	1943	1856	466	87	39	505	26,0
Cycling	896	740	41	156	3	44	4,9
Athletics	488	290	14	198	14	28	5,7
Swimming	219	109	19	110	12	31	14,2
Basketball	190	149	46	41	15	61	32,1
Rugby	175	167	29	8	2	31	17,7
Volleyball	145	71	23	74	23	46	31,7
Water Polo	140	84	24	56	9	33	23,6
Boxing	133	124	14	9	4	18	13,5
Tennis	64	40	10	24	5	15	23,4
Canoe/Kayak	63	36	3	27	1	4	6,3
Rowing	63	42	2	21	0	2	3,2
Fencing	55	29	9	26	6	15	27,3
Shooting	55	36	16	19	6	22	40,0
Handball	54	26	16	28	9	25	46,3
Gymnastics	50	15	7	35	6	13	26,0
Baseball	46	46	22	0	0	22	47,8
Judo	38	28	6	10	1	7	18,4
Archery	37	25	9	12	2	11	29,7
Skating Speed	35	25	5	10	0	5	14,3
Softball	33	0	0	33	6	6	18,2
Wrestling	31	21	10	10	4	14	45,2
Equestrian	30	26	16	4	1	17	56,7
Weightlifting	30	16	9	14	8	17	56,7
Climbing	27	11	1	16	1	2	7,4
Modern Pentathlon	26	15	1	11	0	1	3,8
Hockey Ice	25	25	12	0	0	12	48,0
Karate	22	15	3	7	0	3	13,6
Skiing Alpine	21	13	5	8	1	6	28,6
Field Hockey	20	2	1	18	4	5	25,0

Waterskiing	20	11	5	9	0	5	25,0
Golf	19	11	0	8	1	1	5,3
Lifesaving	19	11	1	8	1	2	10,5
Motorcycle Racing	18	16	2	0	0	2	11,1
Sailing	15	15	1	0	0	1	6,7
Dance Sport	13	6	3	7	5	8	61,5
Taekwondo	13	11	4	2	0	4	30,8
American Football	12	12	8	0	0	8	66,7
Tennis Table	10	6	3	2	0	3	30,0
Automobile Sports	9	9	2	0	0	2	22,2
Bodybuilding	9	4	1	5	0	1	11,1
Casting	9	9	4	0	0	4	44,4
Squash	9	6	1	3	0	1	11,1
Billiards Sports	4	4	3	0	0	3	75,0
Draughts	6	6	2	0	0	2	33,3
Hunting sports	1	1	1	0	0	1	100,0
**TOTAL**	**5340**	**4220**	**880**	**1116**	**189**	**1069**	
**Median**	**30**						

**NO Nicotine**	**TEST**	Males	Females
Badminton	5	3	2
Balle au Tambourin	4	2	2
Biathlon	3	2	1
Bridge	2	2	0
Cricket	2	2	0
Curling	2	2	0
Mixed Martial Arts	4	4	0
Powerboating	2	2	0
Rafting	2	2	0
Skating	31	16	15
Skyrunning	6	3	3
Triathlon	94	60	34
Underwater Sports	43	26	17
**TOTAL**	**200**	**126**	**74**

Year	TEST Sports	Nicotine	%
2016	7042	1421	20,2

**SPORTS**	**TEST**	Males Test	Nicotine Males	Females Test	Nicotine Females	**Total Nicotine**	**Nicotine %**
Soccer	1926	1836	465	90	40	505	26,2
Cycling	1048	835	38	213	4	42	4,0
Athletics	636	388	31	248	15	46	7,2
Basketball	393	344	92	49	15	107	27,2
Swimming	362	194	15	168	8	23	6,4
Volleyball	274	122	45	152	48	93	33,9
Water Polo	261	169	39	92	17	56	21,5
Rugby	187	187	60	0	0	60	32,1
Hockey Indoor	132	127	64	5	0	64	48,5
Handball	131	90	37	41	14	51	38,9
Skiing Alpine	106	58	20	48	6	26	24,5
Underwater Sports	103	50	4	53	3	7	6,8
Hockey Field	101	72	26	29	3	29	28,7
Shooting	96	59	14	37	11	25	26,0
Tennis	91	59	11	32	2	13	14,3
Boxing	90	76	3	14	3	6	6,7
Triathlon	84	57	3	27	0	3	3,6
Hockey Ice	80	71	40	9	3	43	53,8
Rowing	75	49	6	26	0	6	8,0
Weightlifting	67	38	14	29	13	27	40,3
Judo	59	26	3	33	4	7	11,9
Equestrian	55	40	10	15	3	13	23,6
Baseball	51	51	24	0	0	24	47,1
Fencing	49	29	11	20	11	22	44,9
Roller Sports	45	24	4	21	0	4	8,9
Gymnastics	43	14	6	29	3	9	20,9
Diving	39	21	13	18	4	17	43,6
Modern Pentathlon	38	19	1	19	1	2	5,3
Wrestling	36	21	7	15	2	9	25,0
Biathlon	32	16	6	16	0	6	18,8

American football	29	29	15	0	0	15	51,7
Skating Speed	28	14	1	14	1	2	7,1
Life Saving	26	10	1	16	1	2	7,7
Archery	23	9	3	14	5	8	34,8
Dance Sport	22	11	5	11	3	8	36,4
Bodybuilding	18	9	3	9	4	7	38,9
Sport Climbing	17	10	2	7	0	2	11,8
Bridge	16	8	4	8	3	7	43,8
Badminton	15	9	0	6	1	1	6,7
Karate	14	7	1	7	0	1	7,1
Table Tennis	12	8	2	4	0	2	16,7
Skyrunning	10	5	0	5	1	1	10,0
Taekwondo	9	7	1	2	0	1	11,1
Waterskiing	9	6	2	3	2	4	44,4
Casting	8	8	1	0	0	1	12,5
Skiing Mountaineering	8	4	1	4	1	2	25,0
Mixed Martial Art	7	4	0	3	1	1	14,3
Golf	4	4	2	0	0	2	50,0
Powerboating	4	4	2	0	0	2	50,0
Sailing	4	4	3	0	0	3	75,0
Squash	4	4	1	0	0	1	25,0
Kickboxing	3	2	1	1	1	2	66,7
Cricket	2	2	1	0	0	1	50,0
**TOTAL**	**6982**	**5320**	**1164**	**1662**	**257**	**1421**	
**Median**	**39**						

**NO Nicotine**	**TEST**	Males	Females
Boules Sports	3	3	0
Canoe/Cayak	51	28	23
Draughts	2	2	0
Motorcycle Racing	4	4	0
**TOTAL**	**60**	**37**	**23**

Year	TEST Sports	Nicotine	%
2017	8368	1550	18,5

**SPORTS**	**TEST**	Males Test	Nicotine Males	Females Test	Nicotine Females	**Total Nicotine**	**Nicotine %**
Soccer	2879	2726	680	153	44	724	25,1
Cycling	1052	866	52	186	3	55	5,2
Athletics	865	525	37	340	16	53	6,1
Basketball	396	286	63	110	26	89	22,5
Swimming	322	160	2	162	6	8	2,5
Water Polo	239	154	41	85	21	62	25,9
Volleyball	237	96	32	141	32	64	27,0
Rugby	174	170	53	4	0	53	30,5
Handball	155	87	29	68	17	46	29,7
Fencing	116	63	11	53	14	25	21,6
Rowing	110	74	6	36	2	8	7,3
Skating speed	104	49	4	55	7	11	10,6
Hockey Indoor	95	91	30	4	2	32	33,7
Boxing	93	66	7	27	3	10	10,8
Triathlon	88	53	2	35	3	5	5,7
Archery	86	46	17	40	6	23	26,7
Weightlifting	83	42	14	41	18	32	38,6
Gymnastics	81	21	3	60	1	4	4,9
Tennis	75	54	10	21	0	10	13,3
Bodybuilding	72	38	6	34	9	15	20,8
Hockey Ice	70	59	22	11	2	24	34,3
Roller Sports	66	44	10	22	0	10	15,2
Life Saving	61	26	4	35	1	5	8,2
Skiing Cross Country	60	33	6	27	2	8	13,3
Diving	58	30	13	28	7	20	34,5
Skiing Alpine	54	30	12	24	3	15	27,8
Judo	51	29	5	22	3	8	15,7
Baseball	43	43	24	0	0	24	55,8
Shooting	42	20	6	22	6	12	28,6
Underwater Sports	38	24	2	14	1	3	7,9

Canoe/Kayak	34	19	1	15	1	2	5,9
Climbing	30	17	4	13	2	6	20,0
Hockey Field	28	20	6	8	2	8	28,6
Tennis Table	25	13	3	12	1	4	16,0
American football	24	24	9	0	0	9	37,5
Skating Artistic	24	13	0	11	3	3	12,5
Biathlon	23	12	2	11	0	2	8,7
Karate	22	12	1	10	2	3	13,6
Taekwondo	22	11	1	11	4	5	22,7
Equestrian	20	14	7	6	3	10	50,0
Golf	19	11	3	8	6	9	47,4
Wushu	19	19	3	0	0	3	15,8
Automobile Sports	17	17	4	0	0	4	23,5
Tug of War	17	17	2	0	0	2	11,8
Motorcycle Racing	16	16	3	0	0	3	18,8
Powerlifting	14	9	1	5	1	2	14,3
Sailing	12	4	0	8	1	1	8,3
Softball	12	0	0	12	1	1	8,3
Wrestling	10	5	1	5	1	2	20,0
Skiing Mountaineering	9	4	1	5	0	1	11,1
Bridge	8	4	2	4	1	3	37,5
Casting	8	8	1	0	0	1	12,5
Squash	8	6	1	2	0	1	12,5
Skyrunning	7	3	1	4	0	1	14,3
Powerboating	5	5	1	0	0	1	20,0
Waterskiing	5	3	0	2	1	1	20,0
Rafting	4	4	3	0	0	3	75,0
Cricket	2	2	1	0	0	1	50,0
**Total**	**8309**	**6297**	**1265**	**2012**	**285**	**1550**	
**Median**	**40**						

**NO Nicotine**	**TEST**	Males	Females
Badminton	12	6	6
Boules Sports	4	1	3

Curling	4	4	0
Draughts	6	4	2
Kickboxing	10	4	0
Modern Pentathlon	18	9	9
Surfing	5	5	0
**TOTAL**	**59**	**33**	**20**

Year	TEST Sports	Nicotine	%
2018	6737	1023	15,2

**SPORTS**	**TEST**	Males Test	Nicotine Males	Females Test	Nicotine Females	**Total Nicotine**	**Nicotine %**
Soccer	2325	2240	411	85	16	427	18,4
Cycling	783	631	64	152	9	73	9,3
Athletics	540	327	40	213	28	68	12,6
Swimming	350	172	13	178	19	32	9,1
Basketball	308	232	36	76	15	51	16,6
Volleyball	261	140	29	121	33	62	23,8
Rugby	201	189	35	12	0	35	17,4
Water Polo	135	91	18	44	11	29	21,5
Weightlifting	121	64	16	57	8	24	19,8
Triathlon	115	76	5	39	4	9	7,8
Boxing	111	78	6	33	4	10	9,0
Fencing	108	64	11	44	6	17	15,7
Wrestling	83	59	8	24	1	9	10,8
Canoe/Kayak	82	51	3	31	1	4	4,9
Handball	74	37	8	37	6	14	18,9
Skiing Alpine	73	40	8	33	4	12	16,4
Hockey Ice	72	72	13	0	0	13	18,1
Underwater Sports	66	40	3	26	6	9	13,6
Gymnastics	57	27	8	30	5	13	22,8
Rowing	56	44	2	12	0	2	3,6
Diving	55	28	12	27	2	14	25,5
Kickboxing	51	33	4	18	0	4	7,8
Skiing Cross Country	49	25	3	24	2	5	10,2
Biathlon	48	24	0	24	4	4	8,3
Archery	46	23	2	23	2	4	8,7
Life Saving	45	20	3	25	0	3	6,7
Hockey Indoor	40	31	4	9	0	4	10,0
Skating Speed	38	18	2	20	4	6	15,8
Judo	37	15	0	22	4	4	10,8
Equestrian	35	28	6	7	0	6	17,1

Roller Sports	34	20	2	14	0	2	5,9
Shooting	32	19	1	13	1	2	6,3
Hockey Field	24	18	5	6	0	5	20,8
Modern Pentathlon	22	11	0	11	2	2	9,1
Karate	17	10	3	7	0	3	17,6
Mixed Martial Arts	17	13	2	4	0	2	11,8
Snowboard	16	8	3	8	1	4	25,0
Table Tennis	16	10	1	6	0	1	6,3
Taekwondo	16	12	3	4	0	3	18,8
Golf	14	8	1	6	0	1	7,1
Wushu	13	11	1	2	0	1	7,7
Boules_Sports	12	9	3	3	0	3	25,0
Sailing	10	8	1	2	0	1	10,0
Squash	10	9	5	1	0	5	50,0
Tennis	10	10	2	0	0	2	20,0
Bodybuilding	8	4	2	4	0	2	25,0
Dance Sport	8	4	2	4	3	5	62,5
Padel	8	8	3	0	0	3	37,5
Badminton	6	4	1	2	1	2	33,3
Cricket	6	6	1	0	0	1	16,7
Motorcycle Racing	5	5	4	0	0	4	80,0
Casting	4	3	0	1	1	1	25,0
Softball	4	0	0	4	1	1	25,0
**Total**	**6677**	**5129**	**819**	**1548**	**204**	**1023**	
**Median**	**40**						

**NO Nicotine**	**TEST**	Males	Females
American football	10	10	0
Billiards	3	3	0
Chest	7	7	0
Curling	4	4	0
Skyrunning	4	4	0
Polo	8	4	4
Climbing	13	11	2

Fishing	4	4	0
Waterskiing	7	4	3
**TOTAL**	**60**	**51**	**9**

Year	TEST Sports	Nicotine	%
2019	7582	1450	19,1

SPORTS	TEST	Males Test	Nicotine Males	Females Test	Nicotine Females	Total Nicotine	Nicotine %
Soccer	1623	1511	414	112	29	443	27,3
Cycling	862	668	35	194	5	40	4,6
Athletics	851	495	34	356	24	58	6,8
Swimming	420	198	10	222	14	24	5,7
Basketball	289	203	47	86	24	71	24,6
Rugby	282	243	89	39	9	98	34,8
Volleyball	222	115	39	107	44	83	37,4
Water Polo	187	108	28	79	12	40	21,4
Triathlon	181	117	3	64	0	3	1,7
Shooting	163	89	32	74	14	46	28,2
Skating Speed	157	80	8	77	7	15	9,6
Boxing	156	135	22	21	7	29	18,6
Rowing	143	96	6	47	3	9	6,3
Judo	140	86	20	54	5	25	17,9
Gymnastics	131	55	30	76	4	34	26,0
Fencing	122	53	14	69	18	32	26,2
Skiing Alpine	97	64	16	33	0	16	16,5
Skiing Cross Country	86	59	10	27	2	12	14,0
Canoe/Kayak	85	56	7	29	3	10	11,8
Wrestling	84	60	11	24	2	13	15,5
Baseball	72	72	48	0	0	48	66,7
Climbing	72	38	7	34	2	9	12,5
Weightlifting	66	41	18	25	8	26	39,4
Diving	65	35	14	30	3	17	26,2
Tennis	65	36	8	29	2	10	15,4
Archery	62	37	13	25	5	18	29,0
Taekwondo	62	37	9	25	2	11	17,7
Handball	61	29	9	32	9	18	29,5
Underwater Sports	58	40	1	18	5	6	10,3
Hockey Ice	56	56	22	0	0	22	39,3
American football	55	55	23	0	0	23	41,8
Karate	52	36	7	16	3	10	19,2
Biathlon	44	25	7	19	0	7	15,9

Life Saving	42	20	2	22	2	4	9,5
Tennis Table	37	20	3	17	2	5	13,5
Equestrian	33	23	7	10	3	10	30,3
Hockey Field	33	29	16	4	1	17	51,5
Softball	28	0	0	28	9	9	32,1
Bodybuilding	26	17	5	9	2	7	26,9
Snowboard	22	15	5	7	0	5	22,7
Golf	20	16	5	4	2	7	35,0
Boules Sports	18	18	4	0	0	4	22,2
Curling	18	18	7	0	0	7	38,9
Powerlifting	17	14	4	3	1	5	29,4
Hockey Indoor	16	14	1	2	1	2	12,5
Mixed Martial Arts	14	11	1	3	0	1	7,1
Roller Sports	13	3	3	10	3	6	46,2
Skiing Mountaineering	12	6	1	6	1	2	16,7
Squash	11	6	1	5	0	1	9,1
Waterskiing	9	4	3	5	0	3	33,3
Billiards Sports	8	8	6	0	0	6	75,0
Bowling	8	8	2	0	0	2	25,0
Dance Sport	8	3	2	5	2	4	50,0
Ju Jitsu	8	8	2	0	0	2	25,0
Kickboxing	8	4	2	4	2	4	50,0
Skiing Jumping	8	8	3	0	0	3	37,5
Wushu	8	8	2	0	0	2	25,0
Balle au Tambourin	4	4	2	0	0	2	50,0
Powerboating	4	4	2	0	0	2	50,0
Automobile Sports	3	3	2	0	0	2	66,7
**Total**	**7507**	**5320**	**1154**	**2187**	**296**	**1450**	
**Median**	**55**						

**NO Nicotine**	**TEST**	Males	Females
Air Sports	4	4	0
Badminton	13	10	3
Bridge	2	0	2
Chess	2	2	0
Cricket	8	8	0
Draughts	2	2	0

Modern Pentathlon	16	8	8
Motorcycle Racing	2	2	0
Sailing	24	18	6
Surfing	2	2	0
**TOTAL**	**75**	**56**	**19**

Year	TEST Sports	Nicotine	%
2020	2712	539	19,9

SPORTS	TEST	Males Test	Nicotine Males	Females Test	Nicotine Females	Total Nicotine	Nicotine %
Soccer	1126	1020	267	106	27	294	26,1
Cycling	260	194	5	66	1	6	2,3
Athletics	216	135	7	81	1	8	3,7
Volleyball	124	58	22	66	27	49	39,5
Swimming	106	45	2	61	0	2	1,9
Basketball	85	64	11	21	4	15	17,6
Wrestling	72	48	4	24	5	9	12,5
Boxing	62	54	8	8	1	9	14,5
Rugby	60	56	14	4	1	15	25,0
Hockey Ice	36	36	16	0	0	16	44,4
Gymnastics	32	12	7	20	2	9	28,1
Handball	30	18	4	12	3	7	23,3
Water Polo	26	24	8	2	1	9	34,6
Powerlifting	24	15	6	9	0	6	25,0
Skiing Alpine	24	12	3	12	0	3	12,5
Archery	19	7	5	12	5	10	52,6
Motorcycle Racing	19	19	3	0	0	3	15,8
Shooting	18	12	4	6	3	7	38,9
Equestrian	17	17	8	0	0	8	47,1
Dance Sport	16	8	1	8	3	4	25,0
Karate	16	12	4	4	1	5	31,3
Roller Sports	16	8	1	8	0	1	6,3
Diving	14	7	5	7	1	6	42,9
Fencing	12	6	1	6	1	2	16,7
Tennis	12	10	3	2	0	3	25,0
Judo	11	4	2	7	1	3	27,3
Baseball	10	10	8	0	0	8	80,0
Climbing	11	10	4	1	0	4	36,4
Biathlon	8	8	4	0	0	4	50,0
Skiing Mountaineering	8	4	1	4	0	1	12,5
Squash	8	4	1	4	1	2	25,0
Sailing	6	6	2	0	0	2	33,3
American football	4	4	4	0	0	4	100,0

Curling	4	4	1	0	0	1	25,0
Kickboxing	4	2	0	2	1	1	25,0
Tennis table	4	4	2	0	0	2	50,0
Powerboating	2	2	1	0	0	1	50,0
**Total**	**2522**	**1959**	**449**	**563**	**90**	**539**	
**Median**	**16**						

**NO Nicotine**	**TEST**	Males	Females
Canoe/Kayak	5	5	0
Golf	3	3	0
Ju Jitsu	4	4	0
Life Saving	9	8	1
Modern Pentathlon	14	7	7
Rowing	12	8	4
Skating Speed	21	7	14
Skiing Cross Country	20	8	12
Softball	6	0	6
Surfing	4	2	2
Triathlon	77	49	28
Underwater Sports	14	12	2
Weightlifting	1	1	0
**TOTAL**	**190**	**114**	**76**
