# Supplementary material for: Should We be Concerned with Nicotine in Sport? Analysis from 60,802 Doping Control Tests in Italy
Source: Sports Med. 2023 Feb 24;53(6):1273–9. doi: 10.1007/s40279-023-01819-y (PMC9951140; doi:10.1007/s40279-023-01819-y)
Supplement: Supplementary file 2 — Supplementary file2 (PDF 50 KB) [file 40279_2023_1819_MOESM2_ESM.pdf]

Should we be concerned with nicotine in sport?

Analysis from 60,802 doping control tests in Italy

Thomas Zandonai*[1,2,3], Francesco Botrè[4,5], Maria Gabriella Abate[4], Ana María Peiró[2,6,7], Toby Mündel[8,9]

[1] Department of Pharmacology, Paediatrics and Organic Chemistry Miguel Hernández University of Elche, Alicante, Spain

[2] Neuropharmacology on Pain and Functional Diversity (NED), Institute of Health and Biomedical Research of Alicante (ISABIAL Foundation), Alicante, Spain

[3] Department of Social and Developmental Psychology "Sapienza" University of Rome, Rome, Italy

[4] Laboratorio Antidoping, Federazione Medico Sportiva Italiana, Rome, Italy

[5] REDs - Research and Expertise on anti-Doping Sciences, ISSUL Institute des Sciences du Sport, University of Lausanne, Lausanne, Switzerland

[6] Pain Unit, Department of Health of Alicante-General Hospital, Alicante, Spain

[7] Clinical Pharmacology Unit, Department of Health of Alicante - General Hospital, Alicante, Spain

[8] School of Sport, Exercise and Nutrition, Massey University, Palmerston North, New Zealand

[9] Department of Kinesiology, Brock University, St. Catharines, Canada

Thomas Zandonai: ORCID https://orcid.org/0000-0002-7606-9675

Twitter: @thomaszando

Francesco Botré: ORCID https://orcid.org/0000-0001-5296-8126

Twitter: @Botrek

Ana María Peiró: ORCID https://orcid.org/0000-0002-2385-3749

Twitter: @ampeiro

Toby Mündel: ORCID https://orcid.org/0000-0002-4214-8543

Twitter: @kiwiheatlab

Corresponding author:

Thomas Zandonai, PhD

Department of Pharmacology, Pediatrics and Organic Chemistry, Miguel Hernández University of Elche

Alicante, Spain

Address: Crta. Nacional, N-332. s/n, 03550 Sant Joan, Alicante, Spain

E-mail address: tzandonai@umh.es

Supplementary material Table 2.

Nicotine positivity collected In Competition from 2012 to 2020 in Athletics divided into Endurance, Strength-Power and Mixed events.

Year	Sports		Total Tests (n)	Total Nicotine Positive (n)	Nicotine Positivity (%)
2012	Athletics		476	47	9.9
2013	Athletics		704	111	15.8
2014	Athletics		676	84	12.4
2015	Athletics		488	28	5.7
		Endurance	*267*	*7*	*2.6*
		Strength-Power	*86*	*16*	*18.6*
		Mixed	*135*	*5*	*3.7*
2016	Athletics		636	46	7.2
		Endurance	*440*	*18*	*4.1*
		Strength-Power	*142*	*24*	*16.9*
		Mixed	*54*	*4*	*7.4*
2017	Athletics		865	53	6.1
		Endurance	*496*	*22*	*4.4*
		Strength-Power	*305*	*27*	*8.9*
		Mixed	*64*	*4*	*6.3*
2018	Athletics		540	68	12.6
		Endurance	*416*	*41*	*9.9*
		Strength-Power	*99*	*23*	*23.2*
		Mixed	*25*	*4*	*12.6*
2019	Athletics		851	58	6.8
		Endurance	*589*	*33*	*5.6*
		Strength-Power	*222*	*19*	*8.6*
		Mixed	*40*	*6*	*15.0*
2020	Athletics		216	8	3.7
		Endurance	*135*	*5*	*3.7*
		Strength-Power	*58*	*2*	*3.4*
		Mixed	*23*	*1*	*4.3*
